# Natural 3D-Printed Bioinks for Skin Regeneration and Wound Healing: A Systematic Review

**DOI:** 10.3390/polym12081782

**Published:** 2020-08-10

**Authors:** Ali Smandri, Abid Nordin, Ng Min Hwei, Kok-Yong Chin, Izhar Abd Aziz, Mh Busra Fauzi

**Affiliations:** 1 Centre for Tissue Engineering Centre and Regenerative Medicine, Faculty of Medicine, Universiti Kebangsaan Malaysia, Cheras, Kuala Lumpur 56000, Malaysia; alialadil777@gmail.com (A.S.); angela@ppukm.ukm.edu.my (N.M.H.); 2 Department of Physiology, Faculty of Medicine, Universiti Kebangsaan Malaysia, Cheras, Kuala Lumpur 56000, Malaysia; m.abid.nordin@gmail.com; 3 Department of Pharmacology, Faculty of Medicine, Universiti Kebangsaan Malaysia, Cheras, Kuala Lumpur 56000, Malaysia; gabrielchinky@gmail.com; 4 3D Gens Sdn Bhd, 18, Jalan Kerawang U8/108, Bukit Jelutong, Shah Alam 40150, Selangor, Malaysia; izhar@3dgens.com

**Keywords:** 3D-bioprinting, natural-based bioinks, wound healing, skin regeneration, wound dressings

## Abstract

Three-dimensional bioprinting has rapidly paralleled many biomedical applications and assisted in advancing the printing of complex human organs for a better therapeutic practice. The objective of this systematic review is to highlight evidence from the existing studies and evaluate the effectiveness of using natural-based bioinks in skin regeneration and wound healing. A comprehensive search of all relevant original articles was performed based on prespecified eligibility criteria. The search was carried out using PubMed, Web of Science, Scopus, Medline Ovid, and ScienceDirect. Eighteen articles fulfilled the inclusion and exclusion criteria. The animal studies included a total of 151 animals with wound defects. A variety of natural bioinks and skin living cells were implanted in vitro to give insight into the technique through different assessments and findings. Collagen and gelatin hydrogels were most commonly used as bioinks. The follow-up period ranged between one day and six weeks. The majority of animal studies reported that full wound closure was achieved after 2–4 weeks. The results of both in vitro cell culture and in vivo animal studies showed the positive impact of natural bioinks in promoting wound healing. Future research should be focused more on direct the bioprinting of skin wound treatments on animal models to open doors for human clinical trials.

## 1. Introduction

Tissue damage or injury is a severe health problem that annually accounts for around half of the world’s annual health care expenditure [1]. The wound healing mechanism is an immediate protective process that intervenes after the body suffers injury. During this process, damaged or destroyed tissues are disposed of, the vulnerabilities of skin tissues are managed, and skin integrity is restored [2,3]. This process, however, requires excellent patient care and suitable wound coverage. Although traditional wound dressings (i.e., gauze, lint, plaster, and bandages) shield the wound from contaminants, those dressings require frequent changing to avoid neighboring tissue maceration, in addition to their tendency to adhere to the injury, which makes it painful when replacing [4].

Additive manufacturing technologies offer rapid wound treatments to avoid wound contracture and scarring [5]. Three-dimensional bioprinting (3D) is one of the evolving adaptive manufacturing techniques that aim at using biocompatible materials embedded living-cells and growth factors to mimic and restore the natural extracellular matrix (ECM) of human organs [6]. This approach enables the printing of flexible hydrogels layer-by-layer repeatedly through the conversion of computer-aided design (CAD) models into 3D complex structures [7].

Three-dimensional bioprinting involves the fabrication of a complex matrix called bioink [8]. A bioink should be extremely biocompatible to facilitate cell growth, mechanically stable, and should possess high shape fidelity post-printing [9]. Some parameters immensely interfere in determining high functional bioink integrity, including cell-laden parameters (i.e., cell type, cell density, and incubation period), physicochemical properties (i.e., shear-thinning, viscosity, crosslinking degree, and gelation time), and printing parameters (i.e., nozzle temperature and diameter, feed rate, and printing duration) [10,11]. Furthermore, cell selection and sourcing are critical in preventing immune rejection after implantation. Skin primary cells, such as keratinocytes, melanocytes, and fibroblasts, can be appropriately isolated from donor skin and then co-cultured during skin bioprinting applications [12,13]. A variety of natural and synthetic polymer hydrogels were used as bioinks for bioprinting applications. Despite their lack of mechanical stability, 90% of polymers used in bioprinting are derived from natural sources [14]. Natural-based biopolymers have different advantages over synthetic biopolymers, owing to their high similarity with human ECM composition which mimics cells’ native microenvironment to facilitate cell attachment, proliferation, migration, and differentiation [9,15,16].

After introducing 3D bioprinting at the beginning of the last decade, the search for printable and biocompatible polymers became necessary. According to the citation report, the application of a 3D bioprinting approach for wound healing and skin regeneration started in 2012 with the use of collagen bioinks. The number of studies reached 12 studies in 2017 and 19 studies in 2019 to reach a number of around 70 published research in the middle of 2020. Most of the published work introduced natural-based bioinks as a primary or assisted component.

The use of natural polymers in fabricating wound treatments has been the subject of an argument between researchers, and although many of their drawbacks were reported as solvable, no explicit agreement or decision was made. The main objective of this systematic review is to evaluate the effectiveness of using natural-based bioinks as skin substitutes for skin tissue regeneration and wound healing. In addition to reporting the biological properties in both in vitro and in vivo studies, this review further highlights the advances in skin bioprinting and provides potential guidelines for using natural bioinks.

## 2. Methods

### 2.1. Search Strategy

This review was conducted following the preferred notification items for systematic reviews and meta-analyses (PRISMA) checklist [17]. moreover, this review was registered in the prospective international register of systematic reviews (PROSPERO CRD42020167216). A comprehensive search strategy was followed to collect the digital records from five electronic databases: PubMed, Web of Science, Scopus, Medline Ovid, and ScienceDirect. The search was limited to articles published until 1 December 2019. A full update was performed on 1 May 2020.

### 2.2. Search Terms

The search query consists of 18 terms including two sets: (1) skin, “skin regeneration”, “skin tissue engineering”, “Wound healing”, “wound”, and “Burns”; (2) “3D-bioprinting”, “3D bioprinting”, “3D-bio-printing”, “3D printed”, “3D-printed”, “3-D printing”, “3D cell printing”, “Three-dimensional printing”, “Three dimensional printing”, “bioprinting”, “3D scaffold”, and “3D prototyping”. This query aimed at identifying 3D bioprinted skin substitutes as interventions and wound healing or skin regeneration as outcomes.

### 2.3. Study Selection

The reviewers independently screened titles and abstracts of all the identified records for potentially relevant studies. Included records were further reviewed by reading the full text to ensure eligibility. Disagreements were settled through a discussion between authors and, whenever necessary, a third reviewer was consulted. For inclusion, the article should have the following criteria: (1) the use of “natural” bioink(s) for skin; (2) in vitro and in vivo studies; (3) 3D bioprinted scaffold; (4) original article written in the English language only. Articles falling under the following criteria were excluded: (1) particular interest of 3D bioprinting; (2) “synthetic” bioink(s) or crosslinker(s); (3) chronic wounds; and (4) systematic & narrative reviews, interpretations, case series, guidelines, and technical reports.

The following data were recorded from the included studies: (1) study information (authors, publication year, study design, database, and journal name); (2) intervention details (biomaterials and cells used, gelation time, printing temperature, crosslinking materials and methods, and printing techniques); and (3) outcome details (i.e., rheological, mechanical and biological characteristics, wound healing time, and shape fidelity).

### 2.4. Quality Evaluation

The quality of the included studies was assessed following the suggested checklist by the Office of Health Assessment and Translation (OHAT) [18]. The checklist is reported to be applicable to access the potential risk of bias of both in vivo and in vitro studies. This tool considers the following domains: (1) reporting bias, (2) performance bias, (3) detection bias, and (4) selection bias.

## 3. Results

**Figure 1:**
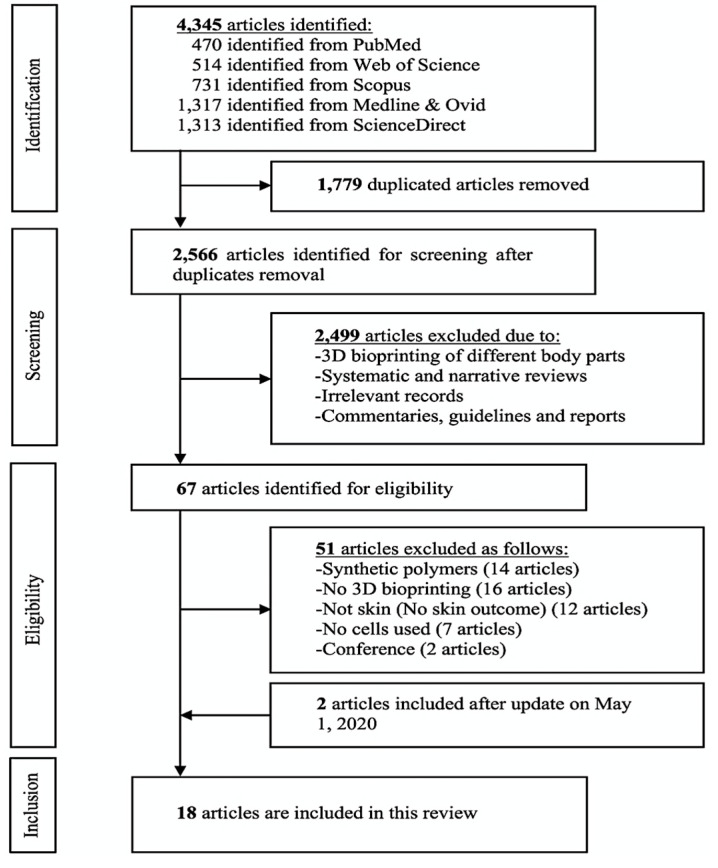
Prisma flowchart of the identified studies.

Initially, the search resulted in 4345 identified articles, and after duplicate removal, 2566 articles were selected for screening. After titles and abstracts screening, 2499 were excluded due to not meeting the inclusion criteria of using natural-based 3D bioprinted skin substitutes for wound healing. The remaining articles were full text screened and updated on May 1, 2020, leaving 18 articles to be included in this systematic review. A flow chart of the search results with reasons for article exclusion is presented in Figure 1.

### 3.1. Included Studies Design

All eligible articles were in vitro and in vivo studies. The data extraction of the included studies is presented in Table 1. The studies were classified depending on the study design, whereas; twelve studies were in vitro [19,20,21,22,23,24,25,26,27,28,29,30], two studies were in vivo [31,32], and four studies conducted both [33,34,35,36]. The characteristics and outcomes of both in vitro and in vivo studies are presented in Table 2 and Table 3, respectively.

### 3.2. Cell and Animal Models

Overall, the majority of in vitro studies used fibroblastic skin cells. Human dermal fibroblasts (HDFs) were commonly used [20,21,22,24,27,28,31,34,35,36], followed by T3T mouse fibroblasts [19,23,25,29,30], and L929 mouse fibroblasts [33,35]. However, human epidermal keratinocytes (HEKs) were also used in four studies [22,31,34,35]. One study used Wharton’s jelly mesenchymal stem cells (WJMSCs) and amniotic epithelial cells (AECs) [26], and another study used epithelial Vero cells [29]. Adipose-derived mesenchymal stem cells (ASCs) and endothelial progenitor cells (EPCs) were also used in one study [34].

For 3D bioprinting in animal studies, the studies included around 151 animal subjects. Each study included 12–40 animals, but one study [34] did not disclose the number of animals used. Four studies reported the use of mice [31,32,33,34], two studies reported the use of rats [35,36], and one study reported the use of porcine [31].

### 3.3. Skin Bioinks

The vast majority of the used wound healing bioinks were gelatin and collagen. Although gelatin hydrogel has high rheological properties, it showed zero viscosity at temperatures above 27 ± 1 °C [21], and all gelatin studies have examined the use of different crosslinking agents [21,23,26,28,32,33,35,36]. On the contrary, four of the six studies reported the ability to print collagen hydrogel without the need for chemical crosslinking agents [25,29,31,34]. The integration of alginate hydrogel with either gelatin [26,28,32] or honey [30] was also reported.

### 3.4. Bioprinting and Crosslinking Techniques

Extrusion-based bioprinting technique was mostly used, and only two studies [22,31] reported the use of inkjet bioprinting technique. Various crosslinking methods were used, and only six studies [25,29,30,31,33,34] reported that no crosslinking agent was applied. The following techniques were used: (1) chemical crosslinking by Ca^+2^ [23,26,27], CaCl_2_ [28,32], 1-ethyl-3-(3-dimethylaminopropyl) carbodiimide (EDC) [24], N-hydroxysuccinimide-1-ethyl-3-(3-dimethylaminopropyl) carbodiimide (EDC-NHS) [19,21,28,36], nebulized sodium bicarbonate (NaHCO_3_) [22], 1,4-butanediol diglycidyl ether (BDDE) [27]; and (2) physical crosslinking by either UV light [20,23,35] or cooling [26,28].

### 3.5. Biocompatibility Measures

Most of the natural-based bioinks were reported to have excellent biological properties. Thirteen of sixteen in vitro studies reported high cell proliferation rates. Even though significant changes in proliferation rate were not evident in three studies [19,22,35], they reported high cell viability. Seven studies reported good cell viability [20,22,24,25,29,30,33], five reported a minimum of 85.07–98% cell viabilities [22,25,26,29,34], and one reported some dead cells indicating low cell viability [24].

Furthermore, fourteen studies reported high cell growth, and only dSIS slurry [24] and SS/GelMA [35] bioinks were found not to facilitate cell growth. All in vivo studies results showed excellent matching with in vitro studies results except for SS/GelMA [35], which showed unique wound healing property after two weeks post-treatment.

### 3.6. Quality Evaluation

The risk of bias of the included studies was conducted using a modified version of the OHAT. In general, the experimental conditions of all reported bioinks were duly mentioned, and almost all studies have low reporting and performance risk of bias. Five of the six in vivo studies have a low risk of bias due to reporting outcome details and fulfilling the selection criteria. Four of twelve in vitro studies showed a low risk of bias as well. In contrast, eight studies have a moderate risk of bias due to the lack of skin cell representation and short follow-up periods, and only one study was found to have a high risk of bias due to high reporting and selection biases (i.e., finding was not clear, adverse events and probability values were not reported; follow-up period, statistical analysis, and outcomes measures were not suitable). The results of the risk assessment are summarized in Table 4.

## 4. Discussion

### 4.1. Overview of the Included Studies

This systematic review shows that natural 3D bioprinted skin substitutes can promote full wound closure based on the pooled results from 18 in vitro cell culture and in vivo animal studies. Most of the 3D bioprinted skin substitutes facilitated cell proliferation, adhesion, and differentiation, and most in vitro studies reported high cell viabilities. Moreover, all animal studies declared total wound area reduction on animals wounded dorsal two weeks post-surgery. However, beyond the limits and practical concerns of evaluating in vitro cell culture and in vivo animal studies and comparing these results to human needs, it must be accepted that animal studies encompass the first level of evidence.

**Figure 2:**
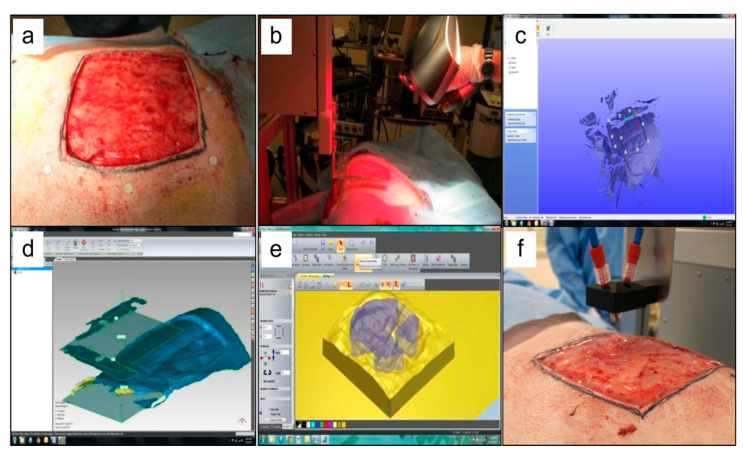
Example of in situ skin bioprinting process, where, (**a**) Markers are placed around the wound area as reference points; (**b**) Wound area scanned with a hand-held ZScanner™ (Z700 scanner); (**c**) Geometric information obtained via scanning is then inputted in the form of an STL file to orient the scanned images to the standard coordinate system; (**d**) The scanned data with its coordinate system is used to generate the fill volume, and the path points for nozzle head to travel to print the fill volume; (**e**,**f**). Output code is then provided to the custom bioprinter control interface for generation of nozzle path needed to print fill volume. Figure and caption reused from Albanna et al. [31]. Used under the Creative Commons License (http://creativecommons.org/licenses/by/4.0/).

The primary objective of using 3D bioprinting in wound healing is to apply the rapid treatment directly to the injured tissues. Albanna et al. have successfully printed fibrinogen and thrombin/collagen I incorporated HFBs and HKCs directly on the dorsal of mice and porcine models (Figure 2). This study resulted in accelerating the process of wound healing in approximately three weeks in comparison to other treatments. The immunohistochemistry study revealed that HFBs and HKCs were found, together with endogenous cells, within the dermis and epidermis layers of the wound 3–6 weeks post-surgery [31].

### 4.2. Bioinks Materials & Combinations

Many types of natural-based bioinks, composite or stand-alone materials, have been proposed to restore the skin integrity and accelerate the wound healing process due to their desirable properties, such as resembling skin ECM, high printability, and excellent biocompatibility as hydrogels are the most commonly used biomaterials [14].

#### 4.2.1. Collagen

Collagen, as a hydrogel, exhibited desirable biodegradability, high shape consistency at 37 °C, and excellent microstructure of micro-and macropores that promote cellular attachment and proliferation [29]. However, collagen direct 3D bioprinting is still limited as collagen solutions have poor printability, especially when incorporated with cells or tissue spheroids [25]. Notably, despite the limited collagen printability, no chemical crosslinking was applied over most of the studies. Instead, this property was overcome by either admixing with other materials such as fibrinogen and thrombin [31], chitosan [19], by using fibrillar collagen [29], by using low concentrations of collagen (2–4%) [25], or by controlling cell suspensions and densities [22]. In the same context, proteins gelation of matrices such as collagen is usually initiated by pH or temperature control or by both. Although this approach is valid for thin structures, it showed diffusion or thermal transference limitations in thick structures (1 to 3 mm), which may lead to the appearance of gelled and non-gelled regions. High levels of pH or temperature may also lead to severe harm to cells [22].

#### 4.2.2. Gelatin

Gelatin is another commonly used bioink that presented high degradability, biocompatibility, and suitable rheological properties. Nevertheless, pure gelatin solutions have weak mechanical strength and low viscosity above 27±1°C, and that limits gelatin usage in 3D bioprinting. It is often mixed with other natural biomaterials, such as alginate [26,28,32] and silk-fibroin [33], to overcome the low formability. Moreover, gelatin methacrylate (GelMA) is also a potential wound healing bioink due to its high thermal sensitivity and photo-crosslinking ability. GelMA is also known to have good biocompatibility, and of promoting cell to cell interaction and cell migration. Furthermore, the advantageous mechanical stability of GelMA after UV crosslinking was used to induce a high shape fidelity of natural-based bioinks, such as cellulose nanofibrils [23] and silk sericin [20].

#### 4.2.3. Alginate

Alginate has been used in different 3D bioprinting applications because of its high shear-thinning and rapid gelation post-printing. However, alginate has many limitations as crosslinking delay may reduce the shape fidelity of the bioprinted constructs, low cell viability as rapid crosslinking limit cell-to-materials interaction. An attempt was conducted by Datta et al. to overcome those limitations by decreasing alginate viscosity using honey to increase cell viability without altering alginate printability. While alginate is qualified for most of the physicochemical properties needed for 3D bioprinting, it suffers poor cell adhesion properties, requiring efforts to enhance the cell adhesion without sacrificing the physicochemical properties [30]. Printing simple alginate solutions were found to have low shape fidelity, although researchers attempted to increase alginate viscosity or extrude it with chemical crosslinkers such as Ca^+2^ [26].

#### 4.2.4. Skin-decellularized Extracellular Matrix (S-dECM)

Extracellular matrix (ECM) represents the non-cellular part of a tissue or an organ, and it mainly assembles the microenvironment network for the cell to perform specific functions. Each tissue has its well-constructed ECM, which consists of several components and proteins that maintain the native structure and support cell migration. Interestingly, the ECM can be derived by using an appropriate protocol and reused as a scaffold for tissue regeneration [37]. Kim et al. successfully decellularized porcine skin-tissue and formed a printable dECM bioink. They found that, in comparison to collagen bioink, the 3D bioprinted skin equivalent using derived ECM bioink promoted dermal compartment stabilization, enhanced epidermal organization, and provided more physiological relevant skin functions in vitro. Moreover, dECM-based 3D skin encapsulated EPCs, and ASCs promoted neovascularization and re-epithelialization as well as wound closure in vivo [34].

### 4.3. Bioink Biocompatibility & Cellular Behavior

Bioinks biocompatibility was duly investigated, and some of the possible reasons that may affect cell viability, adhesion, proliferation, migration, and differentiation were reported. In general, cytotoxicity should be evaluated when proposing a potential material for medical use. Most of the included studies performed MTT assay to ensure no cytotoxicity or inflammation caused by the cell-to-materials chemical interaction. Notably, only silk sericin/GelMA bioink was found to cause acute inflammation on the 7th day, which disappeared at the end of the follow-up period [35].

Bioink pore size should also be considered when choosing a bioink as small pore sizes cause a lack of nutrition and oxygen supply, which led to low cell viability and slower cell migration. Choi et al. studied the effect of gelatin pore size on cell behavior and found that the proliferation rate of HDFs increased by 14% in pore size of 580 μm compared to 435 μm after 14 days [21]. However, using natural bioinks is favorable because of their suitable inter-molecular network. For example, fibrillar collagen is well-known to have a suitable micro- and macropores structure, which was found to highly intervene in increasing cell viability and promoting high cell attachment and proliferation [29].

In the same context, bioink concentration crucially affects cell viability as high concentrations lead to compacted cells. An evaluation of the impact of using different collagen concentration into viscoll on cell viability, found that decreasing collagen concentration from 4% to 2% resulted in increasing the cell viability from 87.2% ± 2.1% to 97.2% ± 1.2% (p < 0.05) [25]. Nocera et al. studied the effect of using smaller collagen extract on NIH 3T3 cell viability and found that decreasing the concentration from 100-extract to 25-extract promoted cell viability from 85.07 ± 6.73% to 111.31 ± 3.65% (p < 0.05) [29]. Xu et al. also studied the effect of admixing small concentrations of GelMA with cellulose nanofibrils (CNFs) on cell proliferation. They found that three days after culture, there was twice the number of cells on CNF/GelMA bioink compared with CNF bioink alone [23].

**Figure 3:**
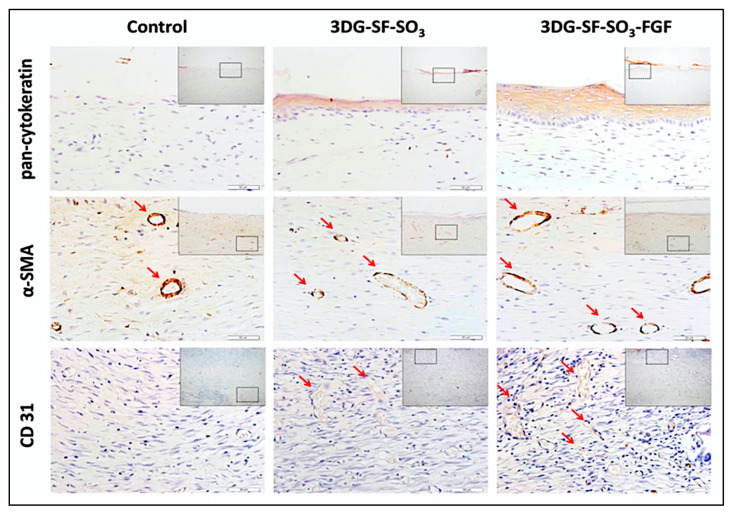
Epidermis and blood vessel formation in skin defects. Immunohistochemical staining of wound sections to detect expression of cytokeratin, SMA and CD31, after implantation with 3DG-SF-SO3 and 3DG-SF- SO3-FGF scaffolds at day 28 post-surgery. Scale bars = 50 μm. Figure and caption reused from Xiong et al. [36]. Used under the Creative Commons License (http://creativecommons.org/licenses/by/4.0/).

On the other hand, growth factors are essential morphogenetic proteins that influence cell activity and direct tissue repair and regeneration [38]. Xiong et al. studied the effect of using a fibroblast growth factor (FGF2) on cell proliferation. They found that adding 100 ng/mL of FGF2 growth factor to the scaffold significantly enhanced the proliferation rate (~40% to ~75%), tissue morphology, and the assembly of the collagen fibril (Figure 3) [36].

Cell suspension densities is another critical factor, as using high densities cause low cell viability. Lee et al. reported the use of an inkjet bioprinting system and studied the effect of using different cell suspension densities and droplet size on cell viability. The study found that cell viability varied proportionally with cell suspension density and inversely with the space between droplets for both keratinocytes (KCs) and fibroblasts (FBs) skin cells. At very low cell suspension density (0.5 million cells/mL) and large droplet spacing (400 mm), FBs cell viability was moderate (84%). Similarly, at a high cell suspension density (5 million cells/mL) and small droplet spacing (400 μm), KCs cell viability was lower (94%) [22]. Moreover, cell adhesion is profoundly affected by the matrix thickness, whereas a higher percentage of cell attachment was observed with 3 mm samples than with 2 mm thick samples. A large thickness scaffold promoted cells to adhere [27].

### 4.4. Structural Design & Mechanical Properties

An effective bioink should possess excellent mechanical properties and should not breakdown post-printing. A bioink should also have high swelling ratios to maintain moisture wound area to exchange nutrients and facilitate cell proliferation. In the literature, human skin was found to have a young’s modulus average of 100 to 1100 kPa [34]. The swelling ratio has an inversible relationship with young’s modulus values, whereas increasing the dSIS filament distances from 500 to 700 µm increased the swelling ratio from 69% to 79% and decreased the Young’s modulus from 26.6 ± 3.8 to 9.7 ± 3.1 kPa (*p* < 0.05) [24]. The same results were reported with CNF crosslinked BDDE [27] and alginate/gelatin [28].

Bioinks should maintain their shape once they leave the tip of the printing nozzle. Overall, proper bioink viscosity ensures high shape fidelity and minimizes the possibility of structural collapse after printing [32]. Shear-thinning is another critical parameter as bioinks should have excellent shear-thinning properties to avoid clogging during the printing process and to regain immediate structural consistency post-printing to be ready to support the next layer [19,26,27]. For example, a period of 1 min was required to ensure the transformation of collagen to gel-state to preserve a solid base for the printing of the next layer [22]. Additionally, the rigidity of the printed scaffolds appeared to affect cell proliferation profoundly. As the rigidity of CNF increases within a tunable range of 3–8 kPa, cell proliferation was promoted [27].

### 4.5. Animal Models & Wound Healing

Early treatment of wounds is critical to avoid wound aggravation and tissue damage over time, due to the hypertrophic scarring. Patients undergoing late treatment often experience severe scarring. Before proposing the capacity of using an effective wound treatment, it must demonstrate high biocompatibility and non-cytotoxicity in vitro. It should also stimulate wound healing and tissue re-epithelization in vivo. Bioprinting human cells resulted in rapid epithelialization represented by 4–5 weeks of acceleration time of wound re-epithelialization [31].

Layering skin constructs in regular pore size and structure significantly influenced nutrition supply and cell ingrowth in the wound area [33]. To ensure scarless wounds, the treatment should be placed evenly in an organized manner on the wound. Xiong et al. reported that the application of the gelatin–sulfonic acid–FGF scaffold on rats’ wounded dorsal helped to smoothen the wound post-surgery, and the cross-sectional results showed complete wound closure in addition to the existence of more blood vessels [36]. Furthermore, the cross-sectional area treated by SS/GelMA showed the formation of new collagen with high fibroblast proliferation similar to the healthy tissue seven days post-surgery followed by complete wound closure on the 4th week, thus proving excellent wound healing properties [35].

For the survival or integration of the new tissue or organ into the surrounding tissue, suitable vascularization is required. Many attempts have been made to build vascularized skin scaffold by using natural-based biopolymers [36] or by printing with interconnective pores sizes between 50 and 500 μm and micropores with diameters lower than 10 μm [29], or by decellularizing skin ECM [34].

## 5. Limitations of the Present Review

This systematic review has several limitations. No specific risk of bias checklist was found to assess in vitro studies. Instead, the OHAT tool was adapted to evaluate both in vitro and in vivo studies. Furthermore, using 3D bioprinting for wound healing is still undergoing animal studies, and no human randomized clinical trials were identified. Another limitation is that the observation time and measurements vary among studies, which causes high heterogeneity in the results. Hence, a meta-analysis was impossible to be performed.

## 6. Conclusions and Future Perspectives

This systematic review identified the potential in vitro cell culture and in vivo animal studies reporting 3D bioprinting skin substitutes. First of all, this review confirms the significant benefits of using 3D printed natural-based bioinks for skin repair and regeneration. Natural bioinks showed excellent ability to mimic the three-dimensional microenvironment structure of native skin tissue and to promote cell adhesion, proliferation, migration, and mobility. Furthermore, in vivo visualization showed full wound closure four weeks post-surgery with well-organized dermal and epidermal layers. This review reported the importance of many bioink properties that should be found to accelerate the wound healing process for a better therapeutic approach. We recommend the use of natural bioinks for wound healing and suggest performing more in vitro studies with the use of a variety of skin cell representations other than dermal fibroblasts, which is known to survive the harsh environment.

Despite the limited number of conducted studies, in situ bioprinting is one of the most promising advances in skin tissue engineering, which can be used by surgeons to print complex organs efficiently and rapidly. Yet, the main challenge is the ability to build tissue details more precisely which required the integration of different fields, including engineering, biology, and medical science. In addition, some new cross-linking techniques, such as two-photon cross-linking and directed on tip UV light, might promote structural control over the existing bioinks. Self-healing hydrogels constitute another interesting direction as they can be printed, retain their pre-vascularized microstructure, and can be used as self-healing scaffolds for wound healing.

## Figures and Tables

**Table 1:** Data extraction of articles’ study design.

Bioinks	Objectives	Study Design	Experimental Design	Cross-linking Method/Materials	Ref.
Collagen-Chitosan blends	Evaluating the rheological and printability of collagen-chitosan composite as a potential bioink.	In vitro	NIH 3T3 cells	NHS/EDC	[19]
CNF/GelMA	Utilizing the use of deficient GelMA concentrations as supporting materials to CNF-based bioink	In vitro	Mouse 3T3 fibroblasts	Ca^+2^ to crosslink CNF UV light to crosslink GelMA	[23]
Sulfated and Rhamnose-rich XRU	Developing polysaccharide modification of 3D bioprinted XRU extract and evaluate its validity.	In vitro	Human dermal fibroblasts (HDFs)	Photo-crosslinking by UV light	[20]
dSIS slurry	Studying the physicochemical and biological properties of dSIS bioink.	In vitro	Normal skin fibroblasts (NSFs)	EDC	[24]
Viscoll Collagen	Evaluating the impact of different collagen concentrations on viscoll to produce high fidelity constructs	In vitro	NIH 3T3	No crosslinking applied	[25]
Alginate/Gelatin	Investigating the rheological behavior of alginate/gelatin as a complex construct.	In vitro	AECs and WJMSCs	Two-steps gelation: a) Gelatin crosslinked by low temperature; b) Alginate crosslinked by Ca^+2^	[26]
BCNFs+ SF/Gelatin	Enhancing the resolution and the mechanical performance of SF/gelatin scaffolds.	In vitro & in vivo	L929 cells & 12 mice	BCNFs work as a crosslinking agent	[33]
Fibrinogen and thrombin/Collagen I	Validating a mobile skin bioprinting system for rapid direct wound management	In vivo	Autologous fibroblasts and keratinocytes& 36 female nude mice + 6 porcine	No crosslinking applied	[31]
CNF	Developing an approach of double cross-linked CNF	In vitro	HDFs	Two-steps gelation: (a) During printing crosslinking with Ca^+2^; (b) Post-printing chemical crosslinking with BDDE	[27]
Sodium Alginate/ Gelatin	Developing dermal skin substitute with controlled structure and adjustable physicochemical properties	In vitro	Human skin fibroblasts (HSFs)	Three-steps gelation: (a) Immediate crosslinking at 4 °C for 30 min; (b) alginate crosslinking by CaCl_2_ for 1 h; (c) crosslinking by EDC-NHS	[28]
Collagen	Developing 3D bioprinted scaffold for tissue engineering application	In vitro	Fibroblastic NIH 3T3, and epithelial Vero cell	No crosslinking applied	[29]
S-dECM	Investigating the ability of printing S-dECM for skin tissue regeneration	In vitro & in vivo	HDFs and HEKs & 8 weeks old male BALB/ cA-nu/nu mice	No crosslinking agent was applied.	[34]
Alginate/Honey	Evaluating the shape fidelity of honey-alginate	In vitro	3T3 fibroblast	No crosslinking applied	[30]
Gelatin	Evaluating the impact of pore size of gelatin scaffold on cell proliferation	In vitro	HDFs	Gelatin was immediately cross-linked by EDC-NHS solution.	[21]
SS/GelMA	Evaluating SS/GelMA bioink for visualization wound care	In vitro & in vivo	L929 cell line, HSF and HaCaT cell lines& 21 female SD rats	The matrices were immediately cross-linked by UV light for 1 min.	[35]
G-SF-SO_3_-FGF2	Fabricating and evaluating porous 3D printed scaffold	In vitro & in vivo	Child foreskin fibroblasts (CFFs)& 36 male Sprague Dawley rats	Post-printing crosslinking, with 1% EDC-NHS solution for 2 h	[36]
Gelatin-Alginate	Studying the effect of 3D-bioprinted gelatin-alginate scaffold on the full-thickness wound healing process	In vivo	40 female mice (6 weeks old)	The gelatin-alginate scaffold was immersed in CaCl_2_ for 10 min	[32]
Collagen	A proof-of-concept study on the ability to print human skin layer-by-layer using a 3D printing system	In vitro	Keratinocytes and fibroblasts	Post printing, nebulized NaHCO_3_ vapor was applied for gelation.	[22]

CNF: Cellulose nanofibrils; GelMA: Gelatin methacrylate; XRU: Xylor-hamnouronic acid; dSIS: Decellularized Small Intestinal Submucosa; Viscoll: A solution of Type I porcine collagen; BCNFs: Bacterial cellulose nanofibers; SF: Silk fibroin; S-dECM: Skin-derived extracellular matrix; SS: Silk sericin; G-SF-SO3-FGF2: Gelatin-sulfonated Silk composite-fibroblast growth factor 2-sulfonic acid group; NHS: N-hydroxy-succinimide; EDC: 1-ethyl-3-[3-dimethylaminopropyl] carbodiimide; BDDE: 1,4-butanediol diglycidyl ether.

**Table 2:** Bioink properties and experimental outcomes of the in vitro studies included.

Bioink	Rheological Properties	Mechanical Properties	Biological Properties	Shape Fidelity	Conclusion	Ref.
Collagen- chitosan blends	Scaffold viscosity: Col/chi 0.36:1 = 1.0 Pa.s Col/chi 0.36:1 = 1.2 Pa.s Col/chi 0.36:1 crosslinked EDC/NHS = 5.6 Pa.s	Elastic modulus: Col/chi 0.36:1 crosslinked EDC/NHS = 1.95 ± 0.14 kPa	(1) Direct cytotoxicity evaluation of Col/chi indicated null toxic effect; (2) Indirect cytotoxicity evaluation suggested that the construct immersion in the medium did not impact the cells either in pure extracts or in 1/16 dilution.	Moderate	Printing different ratios of col/chi under printing flows between 0.19 uL/s and 0.42 uL/s, resulting in acceptable printability values.	[19]
CNF/ GelMA	Scaffold viscosity: CNF/GelMA = 1 × 10^3^ Pa.s swelling ratio: CNF/GelMA (9:10) = 60% CNF/GelMA (2:1) = 60–70% CNF/GelMA (2:1) = 70–87%	Mechanical strength = 2.5–5 kPa Compressive modulus = 2.3–4.5 kPa Surface modulus = 400 to 700 Pa	(1) Promoted the proliferation of fibroblasts; (2) Noncytotoxic and biocompatible features.	High	CNF/GelMA bioink scaffolds showed no cytotoxicity and good cytocompatibility with 3T3 mouse fibroblasts.	[23]
Sulfated & rhamnose rich XRU	Water content: 5% XRU-MA = 98.8% 10% XRU-MA = 96.8% Scaffold viscosity: XRU = 1070.7 Pa.s	Young’s modulus: 5% XRU-MA = ~18 kPa 7.5% XRU-MA = ~153 kPa 10% XRU-MA = ~309 kPa [Increasing the photo-exposure energy from 792 mJ to 2220 mJ increased Young’s modulus of 10% XRU hydrogels from ~182 kPa to ~309 kPa]	(1) Cell proliferation assay on the 10% XRU hydrogels showed a 6.3-fold increase in HDFs cell number two weeks post-culture; (2) Coating XRU with collagen, further promoted cell proliferation with a 7.5-fold increase in cell number 14 days post-culture.	High	When tested with HDFs, XRU hydrogel was found to be extremely compatible with high cell viability and promoted cell attachment and proliferation.	[20]
dSIS slurry	Scaffold viscosity: dSIS slurry = 23.4 Pa.s swelling ratio: P500 = 69% P600 = 74% P700 = 79%	Young’s modulus: P500 = 26.6 ± 3.8 kPa P600 = 17.9 ± 2.6 kPa P700 = 9.7 ± 3.1 kPa	(1) Lower cell adhesion in comparison to control group of spongy scaffolds; (2) Live/dead assay showed only a few dead cells indicating good biocompatibility;	High	The dSIS scaffold developed in the study can be a potential candidate for the application of skin defects with a high level of fidelity and rapid swelling ratio.	[24]
Viscoll collagen	At 25–30 °C: G’ 4%collagen = 1270 ± 138 Pa G’ 3%collagen = 827 ± 41 Pa G’ 2%collagen = 497 ± 13 Pa & G’’ 4%collagen = 416 ± 29 Pa G’’ 3%collagen = 255 ± 20 Pa G’’ 2%collagen = 162 ± 8 Pa	Young’s modulus: 15 mg/mL collagen = 7.2 ± 0.6 kPa 20 mg/mL collagen = 8.2 ± 0.9 kPa 30 mg/mL collagen = 9.5 ± 0.4 kPa 40 mg/mL collagen = 21.5 ± 1.4 kPa	Cell adhesion and proliferation of the bioprinted viscoll scaffold showed good biocompatibility. Cell viability: 4%collagen = 87.2% ± 2.1% 3%collagen = 95.2% ± 1.3% 2%collagen = 97.2% ± 1.2%	High	Enhanced Viscoll bioink allows the creation of contracts of complex geometry without using chemical/photo crosslinking to preserve the predesigned form.	[25]
Alginate/ gelatin	Scaffold viscosity (at 25–40 °C): Alg/gel (2/7.5) = 7 - 4.5 Pa.s Alg/gel (2/10) = 8 - 4.5 Pa.s Alg/gel (2/12.5)= 12.5-7 Pa.s Alg/gel (2/15) = 17.7-7 Pa.s Alg/gel (2/17.5)= 25.5-8 Pa.s	Elastic modulus: Alg/gel (2/7.5) = 280.0 ± 65.7 kPa Alg/gel (2/10) = 230.8 ± 41.4 kPa Alg/gel (2/12.5) = 199.3 ± 14.5 kPa Alg/gel (2/15) = 206.1 ± 11.5 kPa Alg/gel (2/17.5) = 192.3 ± 3.9 kPa 2/15 (alginate/gelatin) indicated: Maximum stress = 554.5 ± 76.1 kPa Maximum strain = 73.1 ± 2.7% Toughness= 106.4 ± 13.3 kJ/m	AECs and WJMSCs proliferated evenly from the 2^ed^ day to 6th day. Bioprinting did not alter the proliferation activity of the two cell types at each predetermined time point. Cell viability: High cell viabilities (>95%) were maintained at day 2, day 4, and day 6.	High	Human AECs demonstrated a superior phenotype of epithelial cells, while WJMSCs exhibited an advanced angiogenic and fibroblastic potential. The presented system of printing alginate/gelatin composite offers promising potential for future skin technology through 3D bioprinting.	[26]
BCNFs + SF/gelatin	When SF/gelatin scaffolds included glycerol, both G’ and G” increased dramatically between 10 min and 20 min.	Tensile modulus = 1.63 ± 0.43 MPa At BCNFs 0.70-PS wt%: Elastic modulus = 186.5 kPa Young’s modulus = 200 kPa	After seven days: L929 cells adhered and proliferated evenly on the silk fibroin/gelatin-BCNFs scaffolds. More importantly, cell viability on BCNFs scaffolds was superior to other groups.	Low	The introduction of nanofibers from bacterial cellulose had a low impact on the printability of the composite bioinks.	[33]
CNF	Swelling degrees: Ca^+2^ crosslinked = 277.7 ± 4.1 Low-level BDDE = 307.1 ± 22.1 High-level BDDE = 212.1 ± 19.4 [After water absorption and drying, the CNF scaffold was able to maintain their shapes].	Young’s modulus: Ca^+2^ crosslinked-CNF = 3.45 kPa Low-level BDDE-CNF = 4.52 kPa High-level BDDE-CNF = 7.44 kPa	3D bioprinted CNF scaffolds showed high cell viability compared with the control 2D cell culture. Compared to the 2D control matrix, cells adhered slightly less on the 3D bioprinting matrix after 12 h of incubation. Three days post cell seeding, the 3D-bioprinted CNF scaffold contains 2 - 4 times more HDFs cells than the 2D control scaffold.	High	3D printing improves the capacity of the produced matrix to promote cell proliferation as opposed to 2D scaffolds, which are essential for rapid wound healing.	[27]
Sodium alginate/ gelatin	Swelling ratio: Alginate/gelatin crosslinked CaCl_2_ = 42% CaCl_2_-EDC = 24% EDC = 301% EDC-CaCl_2_ = 153%	Young’s modulus: Alginate/gelatin crosslinked CaCl_2_ = 175.1 ± 13.3 kPa CaCl_2_-EDC = 240.1 ± 19.9 kPa EDC = 30.6 ± 5.0 kPa EDC-CaCl_2_ = 55.2 ± 4.8 kPa	Both CaCl_2_-EDC and EDC-CaCl_2_ scaffolds promoted HSFs cell proliferation. However, EDC–CaCl_2_ scaffolds were more suitable for cell proliferation than CaCl_2_–EDC in the same environment.	High	Although EDC–CaCl_2_ showed higher cell proliferation, CaCl_2_–EDC was more suitable in terms of physio-chemical and biological properties as a dermal replacement.	[28]
Collagen	Scaffold viscosity: The average viscosity of collagen is 35.62 ± 1.42 Pa.s Swelling ratio: 1437% ± 156%	Not reported.	NIH 3T3 Cell viability: 25-extract = 111.31 ± 3.65% 50-extract = 100.32 ± 1.65% 75-extract = 83.59 ± 6.33%. 100-extract = 85.07 ± 6.73%	High	Fibrillar collagen micro- and macropores structure promoted high cell attachment and proliferation at 37°C.	[29]
S-dECM	Scaffold viscosity (at 15 °C): Collagen = 3 × 10^2^ Pa.s S-dECM = 4 × 10^3^ Pa.s	Young’s modulus: Collagen = 4 kPa S-dECM = 50 kPa	Cell viability: HDFs and HEKs cell viability in both bioinks reached 90% on the 7th day. On the 14th day, cells showed good proliferation in both collagen and S-dMCM.	High	S-dECM bioink could be used to create complex skin constructs by loading different cell types. The fabricated S-dECM bioink showed no cytotoxicity and high biocompatibility, similar to the commercially available collagen type I.	[34]
Alginate/ honey	Scaffold viscosity: A(alginate%)H(honey%) A5H0 = 9.7 ± 0.0 Pa.s A5H1 = 6.2 ± 1.1 Pa.s A5H2 = 6.1 ± 0.0 Pa.s A5H5 = 6.0 ± 0.0 Pa.s A5H10 =5.5 ± 0.1 Pa.s	Tensile strength: A5H0 = 510 kPa A5H1 = 480 kPa A5H2 = 440 kPa A5H5 = 280 kPa	Cell viability: On the 1st day, A5H1, A5H2, and A5H5 scaffolds showed significantly different cell viabilities than A5H0. A5H2 and A5H5 bioinks showed the highest cell proliferation.	High	The 1-2% honey ratio has improved cell proliferation in the bioprinted alginate without a substantial reduction in printability.	[30]
Gelatin	Scaffold viscosity: At 10°C: Gelatin = 450 Pa.s At 30°C: Gelatin = 0 Pa.s [Gelatin viscosity increased remarkably below 27±1°C]	Young’s modulus: G6 (pore size 600 μm) = 98.1 kPa G12(pore size 1200 μm) = 13.7 kPa	HDFs proliferation was 14% higher with pore sizes of more than 580 μm compared to 435 μm in the 3D printed gelatin after 14 days.	High	In G8-G12 gelatin scaffolds, HDFs cell growth rates were approximately 14% higher than in the G6 gelatin scaffold. The mechanical properties were highly dependent on the pore size.	[21]
SS/GelMA	Swelling ratio: SS/GelMA 0.5 = 630% SS/GelMA 0.33 = 495% 20%GelMA = 520%	Not reported.	One the 1st day, L929 cells exhibited a slightly slower growth on SS/GelMA scaffolds of 0.5, 0.33, and 0.2 GelMA in comparison to the control group. While on days 7 and 14 after culture, cell growth was delayed on both matrices and the control group. HaCaT and HSFs cell viabilities were exhibited higher on the scaffolds containing more SS.	High	The inclusion of silk sericin (SS) in the matrices was shown to promote HSFs cell growth. The study also suggested that SS/GelMA is suitable for HaCaT cell culture application as it showed high cell viabilities after seven days.	[35]
G-SF-SO_3_-FGF2	Scaffold porosity: 3DG = ~82.1% 3DG-SF = ~88.0% 3DG-SF-SO3= ~87.6%	The method explained, but no results presented	On the first and third days, similar proliferation rates were noticed by CCK-8 assays with and without FGF2. On the 5th day, proliferation rates were enhanced significantly of almost 40% increase after treating with FGF-2.	High	Using 100 ng/mL of FGF2 led to a ~ 40% higher proliferation rate. Sulfonated SF coated scaffold promoted cell adhesion, proliferation, and growth.	[36]
Collagen	N/A	N/A	Cell viability: FBs = ~98% KCs = ~98%	Low	The study found that FBs and KCs can be evenly printed layer-by-layer as a dermal-like layer and epidermal-like layer. The 3D printing technique provides high dimensional control for engineering skin tissues.	[22]

CNF: Cellulose nanofibrils; GelMA: Gelatin methacrylate; XRU: Xylor-hamnouronic acid; dSIS: Decellularized Small Intestinal Submucosa; Viscoll: A solution of Type I porcine collagen; BCNFs: Bacterial cellulose nanofibers; SF: Silk fibroin; S-dECM: Skin-derived extracellular matrix; SS: Silk sericin; G-SF-SO3-FGF2: Gelatin-sulfonated Silk composite-fibroblast growth factor 2-sulfonic acid group; NHS: N-hydroxy-succinimide; EDC: 1-ethyl-3-[3-dimethylaminopropyl] carbodiimide; BDDE: 1,4-butanediol diglycidyl ether.

**Table 3:** In vivo studies outcomes.

Bioinks	Biological Features	Wound Healing Time	Conclusion	Ref.
BCNFs + SF/gelatin	After seven days, cells could grow under the surface of the printed line at a range of 160-220μm. The hierarchical pore structure of the printed line allowed sufficient space for cell growth.	4 weeks	The findings showed that the arrangement of pore structure is beneficial for nutrient supply for the ingrowth of tissue post-implantation in vivo.	[33]
Fibrinogen and thrombin/Collagen I	One-week post-surgery, the wound area was 66% of the original wound area in contrast to the control group wound area, which remained at 95% (n=12). Two weeks post-surgery, the wound area was 15% of the original wound area, and the control group wound area was 40% (n=8).	10–14 days	In situ 3D bioprinting of autologous cells accelerated the process of wound healing in approximately three weeks in comparison to other treatments.	[31]
S-dECM	Three-weeks post-surgery, S-dECM bioink accelerated wound closure as it consists of different growth factors and cytokines capable of accelerating wound healing. Besides, cells encapsulated dECM accelerated wound re-epithelialization two weeks post-surgery.	3 weeks	Post-implantation, the 3D bioprinted S-dECM bioink enhanced wound closure, neovascularization, and robust blood flow.	[34]
SS/GelMA	The immuno-histochemical observation of IL-6 and TNF-α cytokines indicated acute inflammatory on the 7th day and decreased on the 14th day and hardly found on the 28th day.	2 weeks	Although further in vivo investigations are needed to validate the material, SS/GelMA hydrogel scaffolds represent possible candidates for the application of wound healing and tissue engineering.	[35]
Gel-SF-SO3-FGF2	Two-weeks post-surgery, the epithelial cells tended to migrate from the skin edges towards the wound center in the G-SF-SO3 group. Meanwhile, the dermis and epidermis layers were almost wholly repaired in the 3D G-SF-SO3-FGF group. On the 28th day post-surgery, the wound defect was completely closed in both G-SF-SO3 and G-SF-SO3-FGF2.	2–4 weeks	FGF2 growth factor enhanced the wound healing, re-epithelization as well as promoting blood vessel formation, and expression of various corresponding markers.	[36]
Gelatin-alginate	Post-surgery, the scaffold treatment group showed a significant decline in the wound area. The wound diameter decreased from 0.8 cm on the 1st day to 0.2 cm on the 14th day. The whole wound was nearly healed with almost no crust. On the 14th day, the control group seemed to be covered with hard black crusts, and the mean wound diameter was 0.7 cm. In comparison to the control group, the treatment group formed granulation tissue with uniform and layered wound thickness, which indicates that the scaffold support cell migration and proliferation.	14 ± 1 day	The use of gelatin-alginate was found to decrease wound bleeding and perfusion post-implantation. The scaffold also found to facilitate wound maturation and healing.	[32]

GelMA: Gelatin methacrylate; BCNFs: Bacterial cellulose nanofibers; SF: Silk fibroin; S-dECM: Skin-derived extracellular matrix; SS: Silk sericin; G-SF-SO3-FGF2: Gelatin-sulfonated Silk composite-fibroblast growth factor 2-sulfonic acid group.

**Table 4:** Risk of bias assessment of the included studies.

	References																	
A: Low Risk of Bias B: High Risk of Bias C: Not Clear D: Not Applicable	Heidenreich et al. [19]	Xu et al. [23]	Chen et al. [20]	Shi et al. [24]	Osidak et al. [25]	Liu et al. [26]	Huang et al. [33]	Albanna et al. [31]	Xu et al. [27]	Shi et al. [28]	Nocera et al. [29]	Kim et al. [34]	Datta et al. [30]	Choi et al. [21]	Chen et al. [35]	Xiong et al. [36]	Liu et al. [32]	Lee et al. [22]
Checklist																		
Clear hypothesis/objectives	A	A	A	A	A	A	A	A	A	A	A	A	A	A	A	A	A	A
Clear measures of outcome	A	A	A	A	A	A	A	A	A	A	A	A	A	A	A	A	A	A
Patient characteristics described	B	A	A	A	A	A	A	A	A	A	A	A	A	A	A	A	A	A
Interventions clearly described	A	A	A	B	A	A	A	A	A	A	A	A	A	A	A	A	A	A
Findings were clearly described	A	A	A	A	A	A	A	A	A	A	A	A	A	A	A	A	A	B
Adverse events were reported	B	A	A	A	B	B	B	A	A	B	B	A	A	B	A	A	B	B
Probability values were reported	A	B	B	A	A	A	A	A	A	A	A	A	A	A	B	B	B	B
Exposed and unexposed numbers were matched	C	A	C	B	A	A	B	A	A	C	A	C	A	C	A	A	A	C
Recruitment represents population	A	A	A	A	A	B	A	A	A	A	A	A	A	A	A	A	A	A
Pre-specified and reported outcomes	A	A	A	A	A	A	A	A	A	A	A	A	A	A	A	A	A	A
Participants represent population	B	B	B	B	B	C	A	A	B	B	B	A	B	B	A	A	A	A
Measuring outcomes were blinded	D	D	D	D	D	D	A	A	D	D	D	B	D	D	A	A	A	D
Suitable follow-up period	B	B	B	B	B	B	A	A	B	B	A	B	B	B	A	A	A	B
Appropriate statistical tests	C	A	A	A	A	A	A	A	A	A	A	A	A	A	A	A	A	B
Reliable outcome measures	B	A	A	A	A	A	A	A	A	A	A	A	B	A	A	A	A	B
Groups recruited from the same population	A	A	A	B	A	B	A	A	B	B	A	A	C	C	A	A	A	A
Subjects randomized into intervention	D	D	D	D	D	D	A	A	D	D	D	B	D	D	A	A	A	D
Randomized intervention concealed	D	D	D	D	D	D	A	A	D	D	D	A	D	D	A	A	A	D
Adjustment for confounding	D	D	D	D	D	D	A	B	D	D	D	B	D	D	A	A	A	D
